# No evidence for association between *SLC11A1 *and visceral leishmaniasis in India

**DOI:** 10.1186/1471-2350-12-71

**Published:** 2011-05-20

**Authors:** Sanjana Mehrotra, Joyce Oommen, Anshuman Mishra, Medhavi Sudharshan, Puja Tiwary, Sarra E Jamieson, Michaela Fakiola, Deepa Selvi Rani, Kumarasamy Thangaraj, Madhukar Rai, Shyam Sundar, Jenefer M Blackwell

**Affiliations:** 1 Institute of Medical Sciences, Banaras Hindu University, Varanasi, OS 221 005, India; 2 Telethon Institute for Child Health Research, Centre for Child Health Research, The University of Western Australia, Subiaco, Western Australia, Australia; 3 Cambridge Institute for Medical Research and Department of Medicine, University of Cambridge School of Clinical Medicine, Cambridge, UK; 4 Centre for Cellular and Molecular Biology, Hyderabad, India

**Keywords:** *SLC11A1*, visceral leishmaniasis, genetic susceptibility

## Abstract

**Background:**

*SLC11A1 *has pleiotropic effects on macrophage function and remains a strong candidate for infectious disease susceptibility. 5' and/or 3' polymorphisms have been associated with tuberculosis, leprosy, and visceral leishmaniasis (VL). Most studies undertaken to date were under-powered, and none has been replicated within a population. Association with tuberculosis has replicated variably across populations. Here we investigate *SLC11A1 *and VL in India.

**Methods:**

Nine polymorphisms (rs34448891, rs7573065, rs2276631, rs3731865, rs17221959, rs2279015, rs17235409, rs17235416, rs17229009) that tag linkage disequilibrium blocks across *SLC11A1 *were genotyped in primary family-based (313 cases; 176 families) and replication (941 cases; 992 controls) samples. Family- and population-based analyses were performed to look for association between *SLC11A1 *variants and VL. Quantitative RT/PCR was used to compare SLC11A1 expression in mRNA from paired splenic aspirates taken before and after treatment from 24 VL patients carrying different genotypes at the functional promoter GT_n _polymorphism (rs34448891).

**Results:**

No associations were observed between VL and polymorphisms at *SLC11A1 *that were either robust to correction for multiple testing or replicated across primary and replication samples. No differences in expression of SLC11A1 were observed when comparing pre- and post-treatment samples, or between individuals carrying different genotypes at the GT_n _repeat.

**Conclusions:**

This is the first well-powered study of *SLC11A1 *as a candidate for VL, which we conclude does not have a major role in regulating VL susceptibility in India.

## Background

Visceral leishmaniasis (VL) is a debilitating vector borne disease caused by parasites of the *Leishmania donovani *complex. Prevalence is high in Bihar State in India, indicating a need to understand more about disease pathogenesis to facilitate disease control. Population based epidemiological surveys suggest that 80-90% of individuals infected with *L. donovani *show no clinical symptoms [1,2]. Familial clustering, and the range of clinical outcomes from asymptomatic to fatal disease within and between ethnic groups sharing similar risk factors in Brazil [3,4] and Sudan [5,6], support a contribution of host genotype to susceptibility. Candidate gene and genome-wide linkage studies have highlighted a number of genes/gene regions contributing to disease susceptibility (reviewed [7]). However, replication between study sites has not been observed possibly due to small samples size and limited statistical power.

Amongst the genes studied to date, the most compelling candidate for VL susceptibility is *SLC11A1 *(formerly *NRAMP1*). *SLC11A1 *encodes solute carrier family 11a member 1, a transporter that regulates divalent cation homeostasis in macrophages and has many pleiotropic effects on macrophage activation (reviewed [8,9]). The gene was first identified for its role in controlling *Leishmania donovani *(gene designation *Lsh*), *Salmonella typhimurium *(*Ity*) and *Mycobacterium bov*is (*Bcg*) infections in mice [8,9]. The positionally cloned [10] candidate for these 3 genes was designated as the natural resistance associated macrophage protein 1 (*Nramp1*) for a period before its specific function as a transporter was confirmed [11]. In mice, susceptibility to infection is associated with a coding region mutation in transmembrane domain 4 of *Slc11a1 *[10]. In humans, no functional coding region variants have been identified [12], but common alleles 2 [T(GT)_5_AC(GT)_5_AC(GT)_10_] and 3 [T(GT)_5_AC(GT)_5_AC(GT)_9_] of a functional [13] Z-DNA forming GT_n _repeat (rs34448891) in the promoter region have been associated with disease risk, or protection from, a number of autoimmune and infectious diseases (reviewed [8,14]). Amongst these associations, a common theme was risk of autoimmune disease with allele 3 which drives higher SLC11A1 expression and pro-inflammatory cytokine production by macrophages, while the lower expressing allele 2 was associated with anti-inflammatory cytokines and infectious disease susceptibility, including tuberculosis [15]. In contrast, in Sudan the proinflammatory GT_n _repeat allele 3 is on a risk haplotype for VL caused by *L. donovani *that includes variants at single nucleotide polymorphisms (SNPs) designated 274C/T in exon 3 (rs2276631) and 469 + 14G/C in intron 4 (rs3731865) in the 5' region of the gene [12]. This was interpreted in relation to VL pathology which is associated with a strong pro-inflammatory tumour necrosis factor alpha response [16]. In some populations infectious disease association (listed in additional on-line tables in reference [17]) has been with 3' variants at *SLC11A1 *rather than, or in addition to, the 5' polymorphic loci, including two insertion/deletion (IN/DEL) polymorphisms TGTG/- (rs17235416) and CAAA/- (rs17229009) in the 3'UTR. The latter are potentially regulatory polymorphisms, although this has not been demonstrated experimentally. Here we report on well-powered two-stage primary family-based, followed by a replication population-based, genetic association study that fails to support either a role for polymorphism at *SLC11A1 *in determining susceptibility to VL caused by *L. donovani *in India, or for the GT_n _repeat polymorphism in determining mRNA expression levels of SLC11A1 in splenic aspirates from VL patients before or after chemotherapy.

## Methods

The study was conducted in the district of Muzaffarpur in Bihar State, India, where VL is highly endemic. VL cases and family members or controls were from villages located within a radius of ~100 km from the city of Muzaffarpur covering the districts of Muzaffarpur, Vaishali, Samastipur, Saran, Sheohar, East Champaran and Sitamarhi. Initially, families with at least two siblings affected with clinical VL were ascertained from medical records held in the Kala-Azar Medical Research Centre (KAMRC) in Muzaffarpur, India [18]. This was later extended to collection of singleton cases plus parent (trios) (see Table 1A). The replication study comprised 958 unrelated cases and 1015 unrelated controls. The controls had no history of VL, or a family history of VL among first-, second- or third-degree relatives. Patients and controls were matched for self-reported age, sex, religion, caste and geographic district of recruitment (see Table 1B). Diagnosis of VL was based on presence of typical clinical features of VL i.e. fever with rigors and chills, splenomegaly, weight loss and pancytopenia followed by demonstration of parasites by parasitological methods (light microscopy, *in vitro *culture) using splenic aspirates [19]. Additional VL cases identified in the field were confirmed on the basis of proof of medical records of diagnosis and treatment issued from one of the local health clinics or private practice, and accompanied by demonstration of clinical response to anti-leishmanial treatment (typically with amphotericin B). An annual incidence rate of 2.49 clinical VL cases/1,000 persons has been reported in the region [20]. *L. donovani sensu strictu *(zymodeme MON-2) was confirmed as the causative agent of VL in the study region, in accordance with other reports on clinical isolates from kala-azar patients in the state of Bihar [21-24]. Additional epidemiological and demographic details relating to the study site are described elsewhere [25]. Informed written consent in Hindi was obtained from all participating individuals and from parents of children under 18 years old. Approval for the study was provided by the Ethical Committee of the Institute of Medical Sciences, Banaras Hindu University, Varanasi, India. Collection of families for the primary study was undertaken between 2004 and 2006. The replication study collection was undertaken during 2009-2010. For the family-based primary study DNA was prepared from buccal swabs by whole genome amplification as described [18], and SNPs genotyped using ABI predesigned Taqman assays (ABI, Mulgrave, Victoria, Australia). For the replication case-control study, genomic DNA was extracted from saliva using the Oragene technology (DNA Genotek, Ontario, Canada), and SNPs genotyped using Sequenom iPLEX platform (Sequenom, San Diego, CA). The GT_n _repeat and IN/DELS were genotyped for all samples using ABI fragment analysis processed on an ABI3130 (Australia) or ABI3730 (India) Genetic Analyser.

**Table 1:** Baseline characteristics of (A) families for the primary sample of Indian multicase VL families, and (B) the Indian case-control cohorts.

(A) Family Structure	Number*
N° families	137

N° nuclear families	176

Nuclear families with 1 affected sib	63

Nuclear families with 2 affected sibs	95

Nuclear families with 3 affected sibs	14

Nuclear families with 4 affected sibs	2

Nuclear families with 5 affected sibs	2

N° affected offspring	313

N° affected parents	63

Total N° affected individuals	394

Total N° individuals	836

**(B) Case-Control Sample**	**Number**

**Cases (no.)**	958

Male	571

Female	387

Mean age at study encounter ±SD (yr)	31.2 ±16.7

Range	3-73

Mean age at onset of VL ±SD (yr)	26.8 ±15.3

Religious Group (no.)	
Hindu	850
Muslim	108

**Controls (no.)**	1015

Male	570

Female	445

Mean age at study encounter ±SD (yr)	31.8 ±15.9

Religious Group (no.)	
Hindu	885
Muslim	130

* Numbers are given for the individuals with DNA available for genotyping

Family-based allelic association tests based on the TDT but generalized to allow analysis under additive and genotype-wise models of inheritance were performed within FBAT under the null hypothesis of "no linkage and no association" [26,27]. TDT power approximations [28] show that the 313 primary VL trios had ≥95% power to detect an odds ratio ≥2 at *P *= 0.01 for markers with MAF ≥ 0.1, but only 49% power for an odds ratio of 1.5. Nevertheless, our primary sample was well-powered to detect effect sizes (odds ratios ≥ 2) equivalent to those observed in the earlier study of *SLC11A1 *and VL in Sudan [12]. Robust association tests were performed to take account of multiple trios within a pedigree. Association tests for the replication case-control sample were undertaken using logistic regression analysis performed in PLINK [29] or LOGIT (Stata) using an additive model and a genotypic test. The 941 cases and 992 controls which passed quality control (Hardy-Weinberg Equilibrium) had 100% power to detect associations with an odds ratio of 2 for markers with MAF ≥ 0.1 at *P *= 0.001, and 93.5% power for odds ratio 1.5; MAF ≥ 0.1, *P *= 0.01.

Splenic biopsies were taken as part of routine diagnostic procedure at the Kala Azar Medical Research Centre, Muzaffarpur, Bihar State, India. Since the spleen is a major focus for parasite growth inside macrophages, this afforded an important opportunity to analyse gene expression in a primary site of infection. Pre- and post- treated patient's splenic samples were collected in 5 × RNA Later (Ambion) during 2009-2010, transported to Varanasi at 4°C and stored at -80°C until RNA was isolated. Details regarding age and sex (15 males, median age 16, range 7 to 45 years; 9 females, median age 10, range 8 to 30 years) splenic parasites (21 confirmed positive; 3 not done) and drug administered (19 Miltefosine; 1 Miltefosine + Paramomycin; 1Ambisome + Paramomycin; 3Amphomul) were recorded for each patient. Total RNA was isolated using RNeasy tissue kit (Qiagen) according to the manufacturer's instructions and eluted in 30 ul of RNase free water. Sample quality and integrity was assessed by ND-2000 spectrophotometer (Thermo Fischer Scientific) and agarose (Sigma Aldrich) gel electrophoresis. 500 ng of RNA was reverse transcribed using the High Capacity cDNA synthesis kit (Applied Biosystems). Taqman predesigned gene expression assay (Hs00184453_m1) was used to perform expression studies (7500 HT Real Time PCR system, ABI, Foster City CA, USA) with 18S rRNA (P/N 4319413E) used as an endogenous control to normalize the expression data. Experiments were performed on 24 paired pre- and post-treatment splenic aspirates from VL patients with appropriate no RT and no template controls included in each plate. All samples were run in duplicate. Results were analysed by 7500 software v.2.0.1 and Graph pad prism 5. Paired Student's T tests was used to test for significant differences between pre (Day-0) and post (D-30) expression levels for each genotype, i.e. 3/3, 3/2 and 2/2. One way ANOVA was used to test for differences between 3/3 vs 3/2 vs 2/2 groups at either Day-0 or Day-30.

## Results and Discussion

To undertake our study we initially genotyped 9 polymorphisms (Table 2) in 176 nuclear families (Table 1) used in our previous study [18] that contain 313 offspring with VL collected in the area of Muzaffarpur, Bihar State, India, where *L. donovani *is endemic. This included the putative functional 5' GT_n _repeat and 3'UTR TGTG/- and CAAA/- IN/DELs, as well as the exon 3 274C/T (rs2276631) and intron 4 469 + 14G/C (rs3731865) SNPs shown to be associated with VL in Sudan [12]. Using the family-based association test (FBAT) [30,31] in this primary family dataset (Table 3) we found tentative evidence (nominal *P*-values ≤0.05) for associations between VL and 5' GT_n _repeat, and between VL and the 3'UTR CAAA/- IN/DEL. In particular (Table 3B), homozygosity for the high expressing pro-inflammatory allele 3 at the GT_n _repeat was associated with disease (Z-score = +2.382; nominal *P *= 0.017), while homozygosity for allele 2 (the deletion) was associated with protection for the 3'UTR CAAA IN/DEL (Z-score = -2.332; nominal *P *= 0.019). Since these two markers are not in strong LD with each other (Additional Figure 1: D' = 0.52; r2 = 0.16), these associations are likely to be independent, if real. Neither association is robust to application of a strict Bonferroni correction for 9 SNPs genotyped, which requires a significance cut-off of *P *≤ 0.006 (i.e. *P *= 0.05/9). Given that the 8 SNPs that passed quality control are not all independent (Additional Figure 1), this is over-conservative. A less stringent correction taking account of non-independence of markers provides a cut-off of *P *≤ 0.017 (i.e. *P *= 0.05/3; 2 LD blocks plus 1 independent marker).

**Table 2:** Details of polymorphisms genotyped and the minor allele frequency (MAF) of variants in the Indian study population.

Common Designation	Location	Amino Acid Change	SNP Identity	**Physical Position**^**1 **^**(bp)**	**Alleles**^**2**^	MAF
GTn	5'UTR		rs34448891	218954900	118/120^4^	0.190

-237C/T	5'UTR		rs7573065	218954951	C/T	0.068

274C/T^3^	Exon 3	F66F	rs2276631	218957257	G/A	0.141

469 + 14G/C^3^	Intron 4		rs3731865	218958247	C/G	0.142

823 C/T^3^	Exon 8	G249G	rs17221959	218960874	C/T	0.193

1465-85G/A^3^	Intron 13		rs2279015	218967514	C/T	0.323

D543N G/A^3^	Exon 15	D543N	rs17235409	218967976	G/A	0.073

1729 + 55del4^3 ^(TGTG)	3'UTR		rs17235416	218968058	IN/DEL	0.067

1729 + 263del4^3 ^(CAAA)	3'UTR		rs17229009	218968275	DEL/IN	0.287

^1 ^Physical positions of markers are given according to Build 36.3 of the human genome; ^2^Major > minor alleles for this Indian population; ^3^bp positions of variants relative to an arbitrary site 76bp upstream [33] of the methionine start codon; ^4^allele 3/allele 2 (alleles 1 and 4 were rare or absent from this population).

**Table 3:** Family-based association analysis between *SLC11A1 *and VL

(A) Additive model								
**Common Designation**	**Allele**	**Allele frequency**	**# Fam**	**S**	**E(S)**	**Var(S)**	**Z**	***P***

**GT**_**n**_	2	0.15	68	60	70.38	28.4	-1.947	**0.052**

	3	0.85	68	176	165.62	28.4	1.947	**0.052**

**274C/T**	1	0.88	43	104	99.65	16.3	1.076	0.282

	2	0.12	43	36	40.35	16.3	-1.076	0.282

**469 + 14G/C**	1	0.11	20	17	21.50	9.7	-1.445	0.148

	2	0.89	20	61	56.50	9.7	1.445	0.148

**823 C/T**	1	0.92	46	127	121.97	20	1.127	0.260

	2	0.08	46	41	46.03	20	-1.127	0.260

**1465-85G/A**	1	0.31	92	117	119.63	42.9	-0.402	0.688

	2	0.69	92	229	226.37	42.9	0.402	0.688

**1729 + 55del4 (TGTG)**	1	0.05	19	18	21.50	9.57	-1.131	0.258

	2	0.95	19	54	50.50	9.57	1.131	0.258

**1729 + 263del4 (CAAA)**	1	0.74	88	216	203.33	41.2	1.974	**0.048**

	2	0.26	88	104	116.67	41.2	-1.974	**0.048**

**(B) Genotype-wise model**								

**Common Designation**	**Genotype**	**Genotype frequency**	**# Fam**	**S**	**E(S)**	**Var(S)**	**Z**	***P***

**GT**_**n**_	2\2	0.025	9	8	6.69	2.9	0.759	0.447

	2\3	0.250	68	44	57.00	25.7	-2.565	**0.010**

	3\3	0.725	65	66	54.31	24.1	2.382	**0.017**

**1729 + 263del4 (CAAA)**	1\1	0.566	75	69	63.57	27.9	1.027	0.304

	1\2	0.347	87	78	76.19	33.9	0.311	0.755

	2\2	0.087	26	13	20.24	9.64	-2.332	**0.019**

FBAT analysis under (A) additive and (B) genotype-wise models of inheritance for associations between *SLC11A1 *polymorphisms and the primary family-based sample of VL from Bihar State, India. # Fam = number of families informative for the FBAT analysis; S and E(S) represent the observed and expected transmissions for that allele, Var(S) is the variance. A positive Z score indicates association with disease; a negative Z score indicates the non-associated or protective allele or genotype. Bold indicates significant associations at nominal *P *≤ 0.05. The corrected *P*-value required to achieve significance taking account of multiple but not independent markers is *P *≤ 0.017. Results are shown for the 7 polymorphisms that were genotyped in the primary family-based sample. All passed HWE (*P*-value cut-off 0.05/7 = 0.07 for 7 markers genotyped) in unaffected family founders.

**Figure 1:**
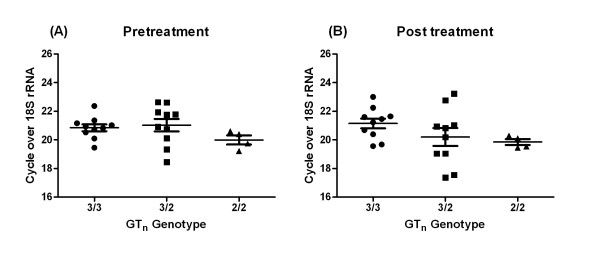
**Relative expression of SLC11A1 mRNA in splenic aspirates from VL patients (A) before (Day 0), and (B) after (Day 30), antileishmanial treatment**. Data are shown according to genotype (3/3, 2/3, 2/2) at the *SLC11A1 *GT_n _promoter repeat polymorphism. Values represent mean ± SEM. Paired Student's T test showed no significant differences in expression of SLC11A1 when D0 values were compared to D30 valules for each genotype. No differences between genotypes were observed at either D0 or D30 as deteremined by one way ANOVA.

Suggestive evidence for an association between Indian VL and allele 3 at the functional promoter region GT_n _polymorphism at *SLC11A1*, which was consistent with data for VL from Sudan [12], prompted us to pursue two further avenues of investigation. First, we looked at expression levels of *SLC11A1 *in mRNA from splenic aspirates from patients carrying the 3 different genotypes at the GT_n _repeat (Figure 1). This failed to show any significant differences in expression levels of SLC11A1 in splenic aspirates from VL patients carrying the three GT_n _genotypes, either before or after treatment, as determined using one way ANOVA. Nor were there differences in expression within each genotype when pre- and post-treatment values were compared using paired Student's T tests. This suggests that differences in expression levels driven by the GT_n _repeat in luciferase assays *in vitro*, especially under lipopolysaccharide and interferon-γ stimulation [13,32], do not necessarily translate into regulation of expression in VL infected spleens *in vivo*. Secondly, we carried out a comprehensive replication of the association study in a much larger population-based case-control sample from the same region of Bihar State in India (Table 4). This study failed to show association between VL and any of the 8 markers that passed quality control. The trend for genotypic association at the GT_n _was in the reverse direction (i.e. a3 was the risk allele in the primary sample, and the protective allele in the replication samples) to that seen in the primary analysis (Table 3). Analyses using caste, which we have shown to provide a good surrogate for population substructure in genome-wide analyses (unpublished data), or religion as covariates also failed to provide evidence for positive associations in the case-control analysis (data not shown).

**Table 4:** Population-based association analysis between *SLC11A1 *and VL

(A) Logistic regression test under additive model							
**Common Designation**	**Allele**	**Affected**	**Unaffected**	**OR**	**L95**	**U95**	***P***

GT_n_	2	355/1473	353/1533	1.05	0.89	1.23	0.585

-237C/T	2	135/1747	122/1862	1.18	0.91	1.52	0.205

274C/T	2	258/1624	286/1698	1.06	0.88	1.27	0.529

469 + 14G/C	1	266/1582	274/1632	1.00	0.83	1.19	0.987

1465-85G/A	1	605/1263	646/1308	1.03	0.90	1.18	0.664

D543N G/A	2	145/1735	112/1526	0.88	0.68	1.14	0.313

1729 + 55del4 (TGTG)	1	110/1488	112/1608	1.06	0.81	1.39	0.669

1729 + 263del4 (CAAA)	2	437/1123	515/1233	0.93	0.80	1.08	0.364

**(B) Genotypic-wise logistic regression analysis (2df)**							

**Common Designation**	**Genotypes**	**Cases**	**Controls**	**OR**	***P***		

GT_n_	2/2	40	27	1	-		

	2/3	275	299	0.62	0.070		

	3/3	599	617	0.66	0.098		

1729 + 263del4 (CAAA)	2/2	67	79	1	-		

	1/2	303	357	0.91	0.346		

	1/1	410	438	0.90	0.583		

Logistic regression analyses under (A) an additive model and (B) using a genotypic (2 df) test for the replication sample of VL cases and controls from Bihar State, India OR = odds ratio (for minor allele relative to major allele in part A); L95 and U95 are lower and upper 95% confidence intervals. In (A) allele counts are shown for mino/major allele for affected and unaffected individuals. There were no significant associations at nominal *P *≤ 0.05. The corrected *P*-value required to achieve significance taking account of multiple but not independent markers is *P *≤ 0.017. Data are shown for all markers that were in HWE (*P*-value cut-off 0.05/9 = 0.006 for 9 markers genotyped) in the control sample. Marker 823C/T failed HWE quality control. Markers D543N G/A (0.1% cases versus 17% controls) and 1729 + 263del4 (CAAA) (17% cases versus 10.7% controls) showed differential missingness between cases and controls, which can be a concern for its potential to generate false positive results [34].

Here we re-examined *SLC11A1 *as a candidate gene for susceptibility to VL in India. Despite preliminary evidence for a role for putative functional polymorphisms in the 5' promoter and 3'UTR regions, we were unable to find supporting evidence for this in functional studies or in a large, well-powered, replication sample for association analysis. We conclude that *SLC11A1 *does not play a major role in determining susceptibility to VL in India. These results also call into question the earlier association observed between VL and SLC11A1 in Sudan [12], which was based on a single small sample of families. Whilst this could represent genetic heterogeneity between human populations and/or the parasite, it is also possible that this first report of association in humans could be a case of beginner's curse. Results presented here suggest that associations observed in small-scale primary samples require validation to determine whether they remain true for the population in which they have been observed.

## Conclusions

This is the first well-powered study of *SLC11A1 *as a candidate for VL, which we conclude does have a major role in regulating VL susceptibility in India.

## Competing interests

The authors declare that they have no competing interests.

## Authors' contributions

AM, MF and SM carried out the field collection and/or preparation of the samples. SM and JO performed the genotyping, and participated in the statistical analysis and interpretation of the data. SEJ trained SM in the laboratory for genotyping techniques, in database entry and use of the genetic database GenIE in Perth, and in genetic statistical analysis methods. MF cross-checked statistical analyses and carried out additional statistical tests. MR oversaw laboratory-based work in Varanasi. DSR and KT oversaw the Sequenom genotyping undertaken by SM in Hyderabad. MS and PT assisted with RNA preparation. SM designed and carried out the QRT/PCR. SS helped conceive the study, was responsible for clinical care of cases at the Kala Azar Medical Research Centre, Muzaffarpur, Bihar State, India, and provided the logistical support to make the study possible. SM prepared the first draft of the manuscript. JMB designed the study, conceived the specific hypothesis to be tested, made the final interpretation of the data, and prepared the final manuscript. All authors read and approved the final manuscript.

## Pre-publication history

The pre-publication history for this paper can be accessed here:

http://www.biomedcentral.com/1471-2350/12/71/prepub

## Supplementary Material

Additional File 1 **Figure S1**. Haploview analysis for D' and r^2 ^pairwise measures of linkage disequilibrium between *SLC11A1 *polymorphisms in the control sample for the replication sample from India. Click here for file
