# Supplementary material for: No evidence for association between SLC11A1 and visceral leishmaniasis in India
Source: BMC Med Genet. 2011 May 20;12:71. doi: 10.1186/1471-2350-12-71 (PMC3128845; doi:10.1186/1471-2350-12-71)

Mehrotra et al.

Additional File 1.

Figure S1. Haploview analysis for (A) D' and (B) r^2 pairwise measures of linkage disequilibrium between *SLC11A1* polymorphisms in the control sample for the replication sample from India. D' values and confidence levels (LOD) are represented as red for D'=1, LOD>2; shades of pink for high D', LOD<2; white for D'<1, LOD<2. r^2 values are represented as black for r^2=1, white for r^2=0, with intermediate values for $0<r^2<1$ indicated by shades of grey. The numbers within the squares represent the D' or r^2 scores for pairwise LD.

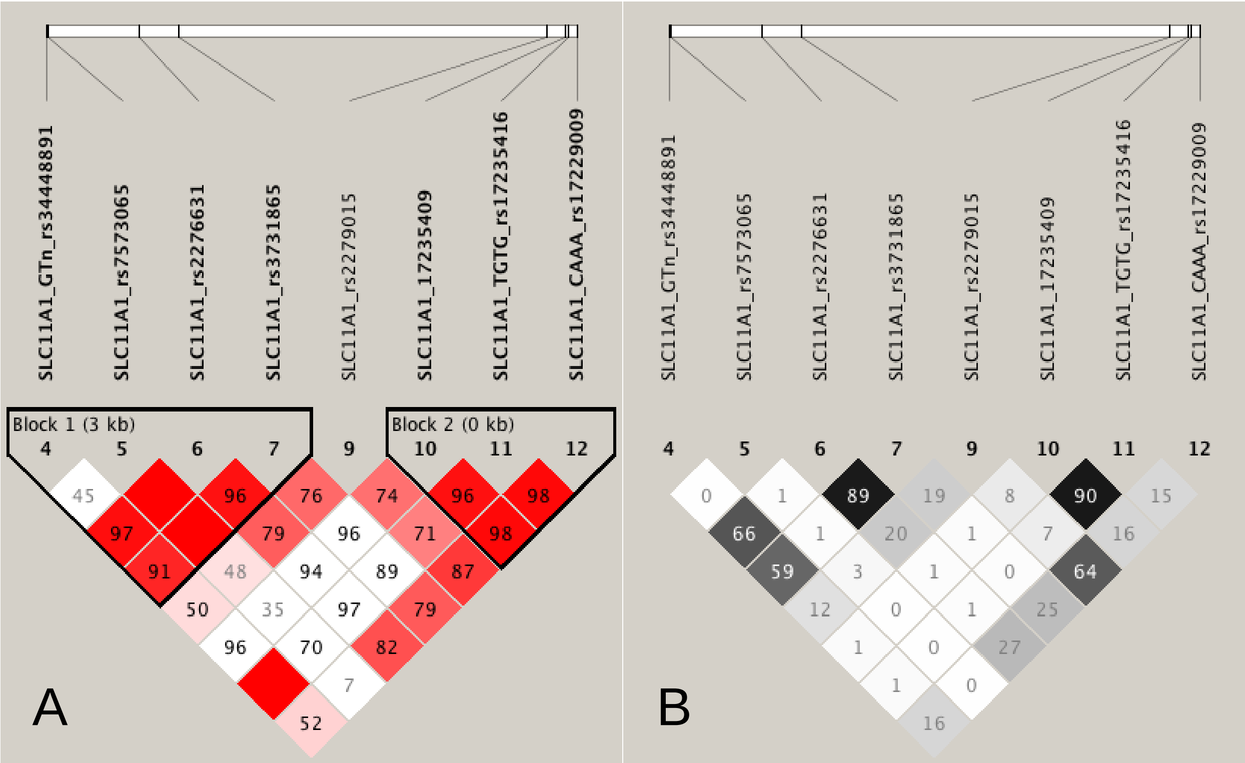

Supplement: Additional File 1 — Figure S1. Haploview analysis for D' and r2 pairwise measures of linkage disequilibrium between SLC11A1 polymorphisms in the control sample for the replication sample from India. [file 1471-2350-12-71-S1.PDF]
